# Fabrication and Optoelectronic Properties of Advanced Quinary Amorphous Oxide Semiconductor InGaZnSnO Thin Film

**DOI:** 10.3390/ma18092090

**Published:** 2025-05-02

**Authors:** Hongyu Wu, Liang Fang, Zhiyi Li, Fang Wu, Shufang Zhang, Gaobin Liu, Hong Zhang, Wanjun Li, Wenlin Feng

**Affiliations:** 1 Chongqing Key Laboratory of Interface Physics in Energy Conversion, College of Physics, Chongqing University, Chongqing 400044, China; wuhy456@163.com (H.W.); 18784063442@163.com (Z.L.); wufang@cqu.edu.cn (F.W.); 2 Center of Modern Physics, Institute for Smart City of Chongqing University in Liyang, Liyang 213300, China; 3 College of AI and Big Data, Chongqing Polytechnic University of Electronic Technology, Chongqing 401331, China; roseymcn2000@foxmail.com; 4 Key Laboratory on Optoelectronic Functional Materials, College of Physics and Electronic Engineering, Chongqing Normal University, Chongqing 401331, China; zhh_2016@163.com (H.Z.); liwj@cqnu.edu.cn (W.L.); 5 Chongqing Key Laboratory of New Energy Storage Materials and Devices, School of Science, Chongqing University of Technology, Chongqing 400054, China; fengwenlin@cqut.edu.cn

**Keywords:** indium gallium zinc tin oxide (IGZTO), magnetron sputtering, transparent conductive oxide (TCO), amorphous oxide semiconductors (AOS), optoelectronic properties

## Abstract

As the typical representative of amorphous oxide semiconductors (AOS), quaternary indium gallium zinc oxide (IGZO) has been applied as the active layer of thin-film transistors (TFTs), but their mobility is still low (usually ~10 cm^2^/Vs). IGTO is reported to have larger mobility owing to the addition of Tin (Sn) in IZO. So, whether Sn doping can increase the optoelectronic properties of IGZO is a new topic worth studying. In this study, four series of quinary InGaZnSnO (IGZTO) oxide thin films were deposited on glass substrates using a high-purity IGZTO (In:Ga:Zn:Sn:O = 1:0.5:1.5:0.25:x, atomic ratio) ceramic target by RF magnetron sputtering. The effects of fabrication parameters (sputtering power, argon gas flow, and target-to-substrate distance) and film thickness on the microstructure, optical, and electrical properties of IGZTO thin films were investigated. The results show that all IGZTO thin films deposited at room temperature (RT) are amorphous and have a smooth and uniform surface with a low roughness (RMS of 0.441 nm, RA of 0.332 nm). They exhibit good average visible light transmittance (89.02~90.69%) and an optical bandgap of 3.47~3.56 eV. When the sputtering power is 90 W, the argon gas flow rate is 50 sccm, and the target-to-substrate distance is 60 mm, the IGZTO films demonstrate optimal electrical properties: carrier concentration (3.66 × 10^19^ cm^−3^), Hall mobility (29.91 cm^2^/Vs), and resistivity (0.54 × 10^−2^ Ω·cm). These results provide a valuable reference for the property modulation of IGZTO films and the potential application in optoelectronic devices such as TFTs.

## 1. Introduction

Since Hosono’s pioneering discovery of amorphous oxide semiconductor (a-AOS) indium–gallium–zinc oxide thin-film transistors (IGZO TFTs) in 2004 [1], amorphous IGZO has been widely recognized for its structural stability, low fabrication temperature, low processing cost, high visible light transmittance, and relatively wide bandgap. These advantages have enabled it to gradually replace hydrogenated amorphous silicon (a-Si:H) and polycrystalline silicon (poly-Si) as the preferred material for large-area and room-temperature-fabricated TFT channel layers. However, with the development of next-generation display technologies, increasing demands on display panel size and performance necessitate TFTs with higher mobility (far exceeding 10 cm^2^/Vs) for driving applications [2]. This imposes higher requirements on the mobility of channel layer materials. Doping has been identified as an effective approach to enhance the mobility of a-AOS films. Kim D.G. et al. reported their selective nitrogen-doped IGZO-TFT using plasma-enhanced atomic layer deposition (PEALD) [3]. Compared with the un-doped one, N-doped IGZO TFTs maintained high mobility (106.5 cm^2^/Vs) while improving stability and uniformity [3]. However, the high fabrication cost associated with this method limits its industrial scalability. For example, 5 at.% Mg doping into IGZO films deposited by sol–gel and spin-coating techniques improved their thermal stability and field effect mobility (0.73~2.35 cm^2^/Vs) [4]. Hf-doped IGZO TFTs prepared through radio frequency (RF) magnetron sputtering by Kim H.G. et al. demonstrated higher mobility (close to 40 cm^2^/Vs) and stability than the un-doped one [5]. In order to substantially improve the field effect mobility and bias stress stability of TFTs, Choi I.M. et al. introduced tin (Sn) into an IGZO target and deposited IGZTO thin films by magnetron sputtering [6]. The mobility of IGZTO TFTs reached 46.7 cm^2^/Vs, twice as much as that of IGZO TFTs (20.3 cm^2^/Vs); the subthreshold (SS) pendulum amplitude of 0.15 V/decade was also lower than the 0.18 V/decade of IGZO TFTs [6]. When introducing Sn^4+^ ions into a-IGZO films, the 5 s energy level of Sn is comparable to that of In, with both contributing to the bottom energy level of the deep conduction band, and with a lower effective mass of the SnO_2_ electrons, there is an opportunity for IGZTO to achieve higher mobility [7]. Therefore, among various doping elements, Sn doping has been identified as the most effective approach to improve and substantially enhance the field effect mobility of IGZO TFTs [6,7,8].

In addition to doping, the quality of the active-layer film is also an important factor to decide the performance of a TFT device. So, some works to improve the properties of IGZO-based TFTs though obtaining high-quality films via suitable preparation process parameters have been reported. For instance, though optimizing the preparation parameters of magnetron sputter-deposited c-axis aligned crystalline (CAAC) IGZO thin film, Zhu B. et al. greatly improved the mobility of IGZO films [9]. Lin T.C. et al. improved the electrical properties of Ga: IZO thin films by tuning the sputtering air pressure and power, with carrier concentrations as high as 1.68 × 10^20^ cm^−3^, average visible transmittance over 87% and mobility up to 47 cm^2^/Vs, and resistivity as low as 7.94 × 10^−4^ Ω cm [10]. Therefore, in order to enhance the performance of IGZTO films, the deposition process parameters, including sputtering power, argon flow, target-to-substrate distances (ToSD), and sputtering gas pressure, need to be optimized. However, the existing literature lacks a systematic and comprehensive investigation of the effects of different fabrication parameters and thickness variations on the properties of IGZTO thin films, which restricts the application and development of IGZTO thin films.

To obtain the optimal fabrication process of IGZTO thin film, IGZTO thin films with different sputtering power, argon flow rate, ToSD, and thickness were deposited by the radio frequency magnetron sputtering method by using an IGZTO ceramic target in this work. The effects of these parameters on the microstructure, optical, and electrical properties of the IGZTO thin films were systematically examined. Additionally, the conduction mechanism of the films was elucidated. This study identifies the optimal fabrication conditions for IGZTO thin films and reveals the trend of their performance as a function of thickness. These findings provide valuable insights for the systematic fabrication and application of IGZTO thin films and lay the foundation for the development of high-performance IGZTO TFTs. These results provide a foundation for the next step of preparing high-performance TFTs and some important reference values for the potential application of IGZTO thin films.

## 2. Experimental Details

### 2.1. Film Preparation

IGZTO thin films were deposited on glass substrates (2 cm × 3 cm) at room temperature (RT) via RF magnetron sputtering. A ceramic IGZTO target (In:Ga:Zn:Sn:O = 1:0.5:1.5:0.25:x, atomic ratio) with a purity of 99.99%, a diameter of 60 mm, and a thickness of 3 mm, being bonded to a 2 mm thick Cu backing plate, was purchased from Zhongshan Zhilong New Materials Technology Co., Ltd. (Zhongshan, China). The deposition was performed using a JGP-450 dual-chamber magnetron sputtering system (Chinese Academy of Sciences Shenyang Scientific Instruments Co., Ltd., Shenyang, China). Prior to IGZTO film deposition, a pre-sputtering process lasting 15 min was conducted to remove the oxide layer and contaminants from the target surface. The substrate maintained rotation with a fixed rotation speed of 12 revolutions per minute (rpm) to enhance the uniformity of the deposited film. During each process of thin film deposition, the base pressure of the background vacuum and the operating pressure were kept at 5 × 10^−4^ Pa and 0.8 Pa, respectively.

Four series of IGZTO thin films were prepared, among which three had the same thickness of 200 nm, but they were deposited under different sputtering powers, argon gas flow rates, or target-to-substrate distances (ToSD); the fourth was prepared under same deposit parameters, but the thickness varied in the range of 100~500 nm due to the different deposition time.

The other detailed deposition parameters for the four series of IGZTO films are listed in Table 1.

### 2.2. Characterization

The electrical properties (carrier concentration, Hall mobility, and resistivity) of the IGZTO thin films were measured by a Hall effect measurement system (Ecopia Corporation Model HMS-3000, Anyang in Gyeonggi-do, Republic of Korea) via the Van der Pauw method. The film sample was 1 cm × 1 cm, and indium (In) metal was used as the electrode. According to the requirements of the Van der Pauw method, four symmetrical electrodes were made at the edge of the sample before the test. The linear I-V relationship indicated that In formed good Ohmic contact with the IGZTO films.

A surface profilometer (Bruker Nano Inc. DektakXT, San Jose, MA, USA) was used to measure the thickness of the films. The optical properties of the films were evaluated using a UV−vis spectrophotometer (Shimadzu UV-3600, Kyoto City, Japan). The surface and cross-sectional morphologies of the films were examined by a scanning electron microscope (Tescan Mira 3 LMH, Brno, Czech Republic) operated at an acceleration voltage of 15 kV. The crystalline phase structure of the films was analyzed using X-ray diffraction (XRD, Rigaku D/max 2500, Tokyo, Japan) with a scanning angle range of 10~80°. The elemental composition and concentration of the films were investigated through X-ray photoelectron spectroscopy (XPS, Thermo Fisher Scientific ESCALAB 250Xi, Waltham, MA, USA). Atomic force microscopy (AFM, Oxford Instruments MFP-3D-BIO, Oxford, UK) was used to observe the surface roughness of the films with a scanning area of 5.0 μm × 5.0 μm.

## 3. Results and Discussion

### 3.1. Structure and Surface Morphology

**Figure 1:**
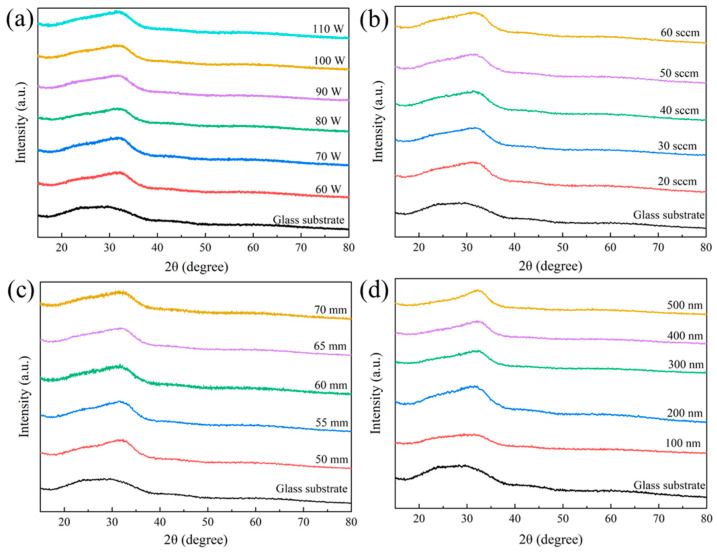
XRD patterns of IGZTO films obtained on glass substrate under different fabrication parameters: (**a**) sputtering powers, (**b**) argon gas flow rates, (**c**) ToSDs, and (**d**) thicknesses.

The XRD patterns of IGZTO thin films prepared under different fabrication parameters (sputtering power, argon gas flow rate, ToSD) and varying thicknesses are presented in Figure 1. In this work, all the film samples were deposited on a glass substrate, so the XRD information includes both the film and substrate. It shows that there exhibits a relatively broad diffraction peak in the 2θ range of 20° to 35° for all films and glass substrates, in which some results from the intrinsic diffraction peak of the glass substrate used in the experiment [11]. But the peak intensity in the 30–35° of IGZTO films is found to be observably stronger than that of the glass substrate, which means the enhancement in peak intensity originated from the films. Chang et al. demonstrated through TEM that the weak broad peak of IGZO thin films at 33–34° can be attributed to low-density and small-sized nano-crystallites [12]. The amorphous phase of IGZO thin films is not entirely amorphous but rather an amorphous phase with local small crystallites [13]. A similar phenomenon is observed in our IGZTO thin films, meaning that the broad diffraction peak observed at 33° is not attributed to any specific phase of the IGZTO thin film. Therefore, the IGZTO thin films prepared in this study exhibit an amorphous structure, indicating that the variation of fabrication parameters and thickness does not affect the phase structure of the IGZTO thin films.

**Figure 2:**
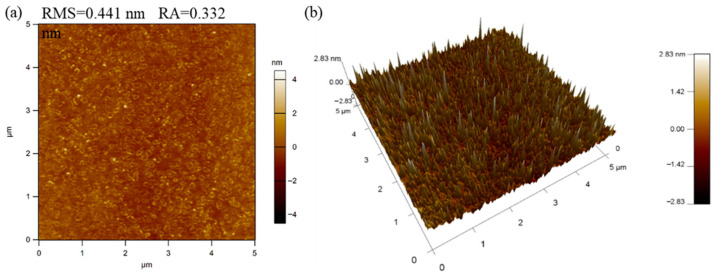
AFM images of a 200 nm−thick IGZTO thin film prepared at a sputtering power of 90 W, argon gas flow rate of 50 sccm, and ToSD of 60 mm: (**a**) surface topography and (**b**) 3D surface topography.

Figure 2 shows the AFM surface morphology of a 200 nm-thick IGZTO thin film prepared at a sputtering power of 90 W, argon gas flow rate of 50 sccm, and ToSD of 60 mm. The arithmetic mean roughness, i.e., the roughness average (RA) and root mean square roughness (RMS) of the IGZTO thin film, was calculated to be 0.332 nm and 0.441 nm, respectively. These low roughness values indicate a uniform and high-quality surface. The smooth surface of the IGZTO thin film, characterized by its multicomponent and amorphous nature, is conducive to higher visible light transmittance [14].

### 3.2. Optical Properties

#### 3.2.1. Transmittance Spectra

**Figure 3:**
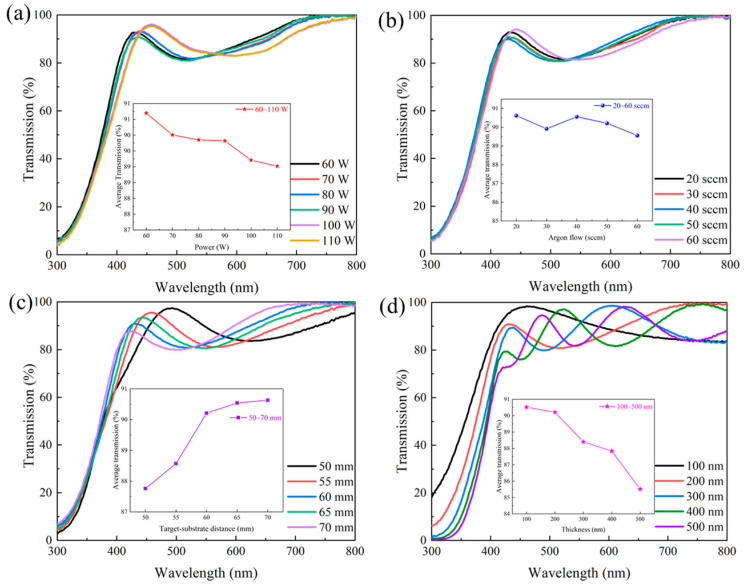
Optical transmittance curves of IGZTO films obtained under different deposit parameters: (**a**) sputtering powers, (**b**) argon flow rates, (**c**) ToSDs, and (**d**) thicknesses. (The insets show the average visible transmittance in the 400~800 nm band.)

Figure 3 is the transmittance spectra of IGZTO thin films in the wavelength range of 300–800 nm and the average visible light transmittance. To eliminate the influence of substrate reflection and absorption on the measurement results, a blank glass slide was used for baseline calibration. The average transmittance of IGZTO thin films in the visible light region under different sputtering powers, argon gas flow rates, ToSDs, and film thicknesses was calculated using Equation (1):

(1) $$T_{average}=\frac{\int_{\lambda_{1}}^{\lambda_{n}}T(\lambda )d\lambda }{\lambda_{n}-\lambda_{1}}\approx \frac{1}{m}\sum_{\lambda =\lambda_{1}}^{m}T\left(\lambda \right)\;(m=\lambda_{1},\lambda_{2},\lambda_{3}\ldots \lambda_{n})$$

where *λ*_1_ = 400 nm and *λ_n_* = *λ*_2_ = 800 nm in this work.

Figure 3a shows how the average visible light transmittance of IGZTO thin films gradually decreases with increasing sputtering power from 60 W to 110 W but remains above 89%. This indicates that IGZTO thin films exhibit very low absorption in the visible and near-ultraviolet regions. From Figure 3b, it is observed that the average visible light transmittance of IGZTO thin films does not follow a clear trend within the argon gas flow rate range of 20~60 sccm, with values of 90.62%, 89.92%, 90.56%, 90.21%, and 89.56%, respectively.

As depicted in Figure 3c, the average visible light transmittance of IGZTO thin films increases with the ToSD. Specifically, when the ToSD is increased from 50 mm to 60 mm, the average visible light transmittance rapidly rises from 89.76% to 90.21%. Further increasing the ToSD from 60 mm to 70 mm results in a gradual increase in transmittance to 90.63%. For ToSDs ≥ 60 mm, the average visible light transmittance of the films exceeds 90%. The interference fringes observed in the transmittance curves are attributed to the discontinuities at the interfaces between air and the film, as well as between the film and the glass substrate [15].

As shown in Figure 3d, in the visible light region of 400~800 nm, only the 100 nm thick IGZTO film does not exhibit interference fringes. All films maintain a visible light transmittance above 85%. With increasing film thickness from 100 nm to 500 nm, the visible light transmittance decreases from 90.52% to 85.51%. This decline is primarily due to the enhanced absorption and reflection of light as the film thickness increases. These results demonstrate that IGZTO thin films possess excellent visible light transmittance, making them highly promising for applications in display technologies as transparent conductive materials [16].

#### 3.2.2. Optical Bandgap

The optical bandgap width ($E_{g}$) of IGZTO thin films can be determined based on their transmittance. The relationship between $E_{g}$ and the sputtering power, argon gas flow rate, ToSD, and film thickness is established as follows: As a direct bandgap semiconductor [17], the absorption coefficient (α) of the IGZTO films can be calculated from the transmittance using Equation (2). The $E_{g}$ is subsequently derived using Equation (3) [18]:

(2) $$\alpha =-\frac{1}{d}ln(T)$$

(3) $${\left(\alpha hv\right)}^{2}=C(hv-E_{g})$$

The $E_{g}$ of IGZTO thin films was determined from the linear portion of the plot of (αhv)^2^ versus hv, where α is the optical absorption coefficient, d is the film thickness, T is the transmittance, hv is the photon energy, and C is a constant.

**Figure 4:**
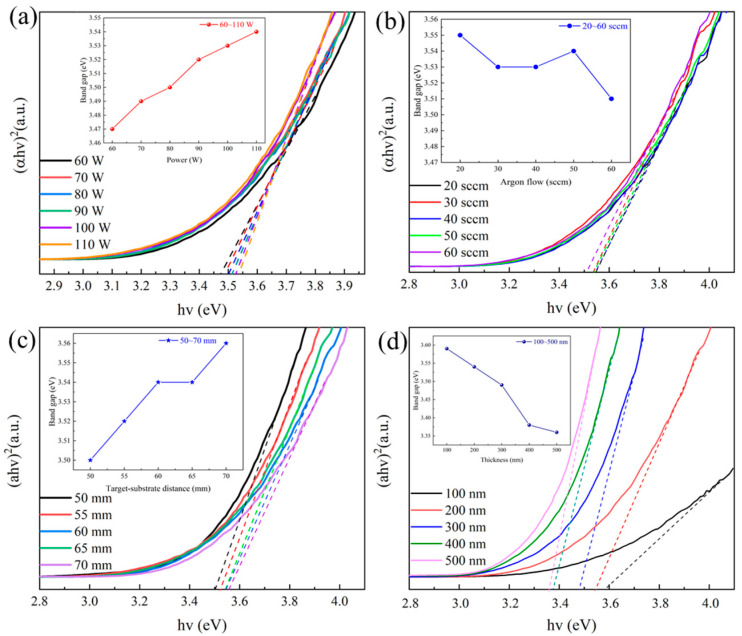
Plots of (αhv)^2^ versus hv for IGZTO thin films deposited under different parameters: (**a**) sputtering powers, (**b**) argon gas flow rates, (**c**) ToSDs, and (**d**) film thicknesses (the insets show the corresponding variations in $E_{g}$).

Figure 4a shows the relationship between sputtering power and the $E_{g}$ of IGZTO thin films. The $E_{g}$ of IGZTO thin films increased linearly with increasing sputtering power. Specifically, the $E_{g}$ increased from 3.47 eV to 3.54 eV as the sputtering power was raised from 60 W to 110 W. This trend can be attributed to the increase in carrier concentration in IGZTO thin films with increasing sputtering power. According to the Burstein–Moss theory [19,20], an increase in carrier concentration shifts the Fermi level in degenerate semiconductors, resulting in an increase in the $E_{g}$ of the films.

The relationship between the argon gas flow rate and the $E_{g}$ of IGZTO thin films is illustrated in Figure 4b. It reveals that unlike the trend observed with sputtering power, the $E_{g}$ did not exhibit a clear linear relationship with the argon gas flow rate. When the argon gas flow rate was 20 sccm, the high energy of the sputtered particles led to a higher crystallinity of the IGZTO thin films, resulting in a higher $E_{g}$ of 3.55 eV. As the argon gas flow rate increased to 50 sccm, the energy of the sputtered particles decreased, reducing the crystallinity of the films. However, the films became smoother and denser, with a corresponding decrease in $E_{g}$ to 3.54 eV. Further increasing the argon gas flow rate to 60 sccm resulted in a lower energy of the sputtered particles, which led to an increase in grain boundaries and defects in the films, thereby reducing the $E_{g}$ to 3.51 eV.

Figure 4c presents the relationship between ToSD and the $E_{g}$ of IGZTO thin films. The $E_{g}$ increased with increasing ToSD. When the ToSD was 50 mm, the higher kinetic energy of the sputtered particles led to a faster deposition rate of the IGZTO thin films. The films reached a thickness of 200 nm more rapidly, but this also resulted in more defects and larger grain sizes, which weakened the quantum size effect and led to a lower $E_{g}$ of 3.50 eV [21]. In contrast, when the ToSD was increased to 70 mm, the deposition rate decreased, and the energy of the sputtered particles arriving at the glass substrate was reduced. Consequently, the migration energy of the films on the substrate surface decreased, resulting in smaller grain sizes and an enhanced quantum size effect. This led to an increase in the $E_{g}$ to 3.56 eV.

The thickness dependence of $E_{g}$ is given in Figure 4d. It shows that $E_{g}$ decreases from 3.59 eV to 3.36 eV as the film thickness increases from 100 nm to 500 nm. This decrease is attributed to the fact that the carrier concentration in thicker film increases, leading to the aggregation of more carriers, which promotes the electron jump from the valence band to the conduction band [22], and that the increase in carrier concentration in wide bandgap semiconductor films enhances electron–electron and electron–ion interactions as well as impurity scattering, which resulting in a narrowing of the bandgap [23].

The trend of decreasing $E_{g}$ became more stabilized as the thickness increased from 400 nm to 500 nm. In contrast, the $E_{g}$ of films with thicknesses ranging from 100 nm to 400 nm exhibited more pronounced changes with thickness, which can be attributed to the quantum confinement effect within the thin film layers [24,25,26].

#### 3.2.3. Urbach Energy

From the absorbance of a film, the Urbach energy ($E_{u}$), which can describe the optical disorder of the thin film, can be calculated. There is a relationship between the absorption coefficient and $E_{u}$ as follows [27]:

(4) $$\alpha =\alpha_{0}\;exp\left(\frac{hv-E_{g}}{E_{u}}\right)$$

where α is the absorption coefficient, hv is the optical quantum energy, and $E_{u}$ denotes the Urbach energy, which is commonly characterized as the extent of broadening in the tail of localized states within the band gap.

Equation (4) can be changed to the following form:

(5) $$\ln\alpha ={ln\alpha }_{0\;}+\frac{1}{E_{u}}(hv-E_{g})$$

From Equation (5), it shows that$\;\frac{1}{E_{u}}$ is the slope of the straight lnα-hv line, indicating that $E_{u}$ can be calculated from the reciprocal of the slope of the linear part of the lnα-hv line.

**Figure 5:**
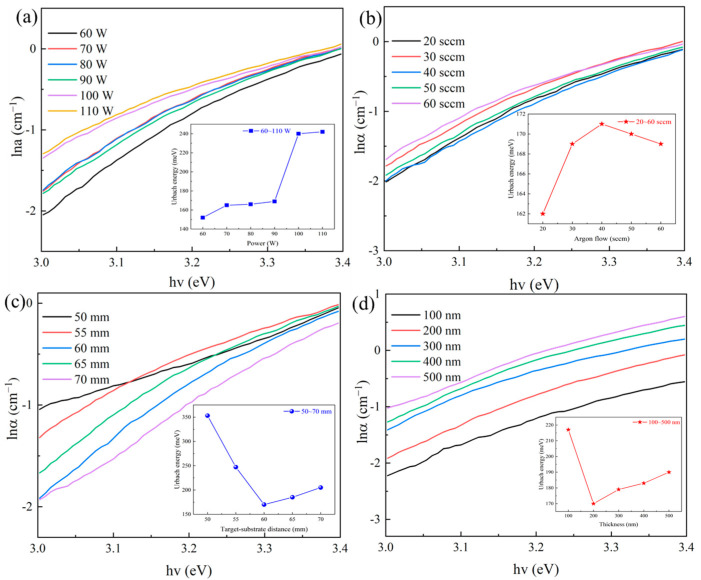
Plots of ln (α) versus hv for IGZTO films deposited at different parameters: (**a**) sputtering powers, (**b**) argon gsa flow rates, (**c**) ToSDs, and (**d**) film thicknesses (the insets show the corresponding variations of $E_{u}$).

The lnα-hv lines and the calculated $E_{u}$ of IGZTO films deposited at different preparation process parameters are demonstrated in Figure 5.

It can be observed from Figure 5a that when the sputtering power is increased from 60 W to 110 W, the $E_{u}$ of the IGZTO films enhances from 152 meV to 242 meV, indicating an increase of the internal structural disorder of the films. The larger Urbach energy is attributed to the decrease in film quality, i.e., larger disorder, or more defects present in the films [28,29]. At sputtering powers of 60~90 W, the $E_{u}$ of the films is between 152 and 169 meV, with lower disorder and higher quality of the films. When the sputtering power exceeds 90 W, the concentration and speed of the sputtered particles increase, and the film grows too fast, leading to a great number of grain boundaries and defects in the film, with the $E_{u}$ exceeding 240 meV.

From Figure 5b, it shows that the $E_{u}$ of the film increases firstly and then decreases with the increase in the Ar flow rate, and the highest $E_{u}$ (171 meV) is obtained at a 40 sccm Ar flow rate. The IGZTO films obtained at low Ar flow rates (<40 sccm) will achieve fewer defects. The $E_{u}$ of all films prepared at different Ar flow rates is in the small range of 162~171 meV, which indicates that the impact of the Ar flow rate on better quality is not large.

From Figure 5c,d, it is found that the $E_{u}$ first decreases but then increases with the increase in the ToSD or film thickness. The smallest $E_{u}$ of 170 meV occurs at 60 mm ToSD or 200 nm thickness, respectively. When the ToSD is 50~55 mm, the sputtering particle rate is fast and the energy is large, which may cause secondary bombardment to the deposited film, and the quality of the film is reduced and more defects are generated, resulting in an $E_{u}$ as high as 353 meV. When the ToSD is 65~70 mm, the particle energy loss increases, the deposition energy decreases, the film growth is limited, the grains become smaller, the grain boundaries increase, and the defects increase, resulting in a gradual increase of $E_{u}$ to 205 meV. When the thickness is 100~200 nm, the thicker films can form a complete crystal structure and reduce the defects on the surface and interface, which cause a decrease in $E_{u}$. When the thickness is 200~500 nm, the internal stresses of the films increase, which may produce lattice distortions, leading to an increase in defects and $E_{u}$ gradually increasing to 190 meV.

### 3.3. Electrical Properties

#### 3.3.1. The Effect of Sputtering Power

**Figure 6:**
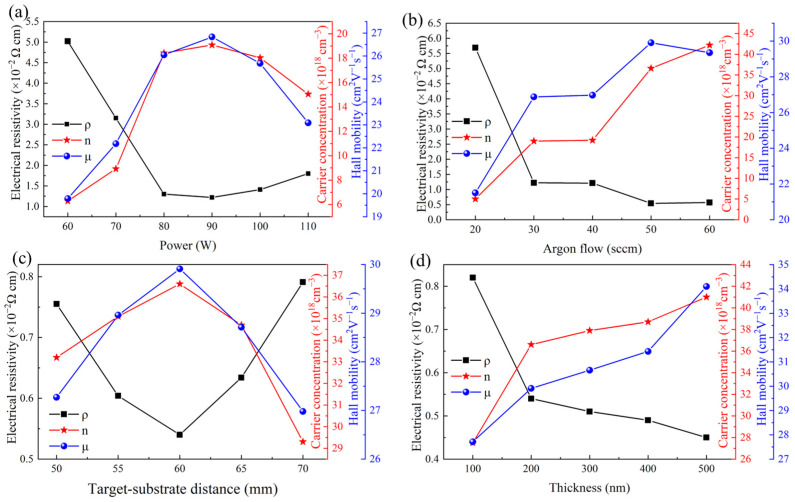
Variation of resistivity, Hall mobility, and carrier concentration in IGZTO thin films deposited at different parameters: (**a**) sputtering powers, (**b**) argon gas flow rates, (**c**) ToSDs, and (**d**) film thicknesses.

Figure 6a shows the electrical properties of IGZTO thin films prepared at different sputtering powers, including resistivity, Hall mobility, and carrier concentration. When the sputtering power was 60 W, the low number and energy of sputtered particles resulted in a slow deposition rate and poor film quality with low density. Consequently, the electrical performance was inferior, with a Hall mobility of 19.79 cm^2^/Vs, a carrier concentration of 6.29 × 10^18^ cm^−3^, and a resistivity of 5.02 × 10^−2^ Ω·cm. As the sputtering power increased to 90 W, a higher number of energetic argon ions bombarded the target surface, increasing the deposition rate of IGZTO thin films. This enhanced rate required more oxygen atoms to combine with the target particles, leading to an increase in oxygen vacancies [30].

Additionally, the higher kinetic energy of the sputtered particles enabled more extensive diffusion and migration on the substrate surface, resulting in denser and higher-quality films. Consequently, the Hall mobility increased to 26.83 cm^2^/Vs, the carrier concentration rose to 1.91 × 10^19^ cm^−3^, and the resistivity decreased to 1.22 × 10^−2^ Ω·cm. When the sputtering power was further increased to 100 W and above, the higher energy of the sputtered particles began to erode the already well-formed IGZTO films, degrading their density and smoothness. This led to an increase in grain boundaries and defects, resulting in a decrease in carrier concentration to 1.51 × 10^19^ cm^−3^, a reduction in Hall mobility to 23.09 cm^2^/Vs, and an increase in resistivity to 1.80 × 10^−2^ Ω·cm.

In summary, the optimal electrical properties of IGZTO thin films were achieved at a sputtering power of 90 W in this work.

#### 3.3.2. The Effect of Argon Gas Flow Rates

The electrical properties of IGZTO thin films prepared at different argon gas flow rates are presented in Figure 6b. It shows that at an argon gas flow rate of 20 sccm, the electrical performance of the IGZTO thin films was relatively poor, with a Hall mobility of 21.50 cm^2^/Vs, a carrier concentration of 5.0 × 10^18^ cm^−3^, and a resistivity of 5.69 × 10^−2^ Ω·cm. When the argon gas flow rate was increased to 50 sccm, the Hall mobility of the IGZTO thin films increased to 29.91 cm^2^/Vs, the carrier concentration rose to 3.66 × 10^19^ cm^−3^, and the resistivity decreased to 0.54 × 10^−2^ Ω·cm. Further increasing the argon gas flow rate to 60 sccm resulted in a higher carrier concentration of 4.22 × 10^19^ cm^−3^. However, the Hall mobility decreased to 29.35 cm^2^/Vs, and the resistivity increased to 0.57 × 10^−2^ Ω·cm.

It is known that carriers mainly come from oxygen vacancies in oxide film. The more oxygen vacancies, the larger the carrier concentration. When the argon gas flow rate increases, the oxygen level in the sputter atmosphere becomes relatively lower, so the oxygen vacancies in films will increase, leading to a higher carrier concentration. Thus, the carrier concentration in IGZTO film increase with the argon gas flow rate.

The influence of the argon gas flow rate on mobility can be explained by the change in the mean free path. The mean free path (λ) of the IGZTO film at different argon flow rates can be calculated by Equation (6):

(6) $$\lambda =\frac{K_{B}T}{\sqrt{2}\pi Pd^{2}}\;$$

where d is the diameter of the molecule; $K_{B}$ is Boltzmann’s constant; T is the temperature; P is the pressure. From Equation (6), it shows that the larger the P, the smaller the λ.

When the argon gas flow rate is small, i.e., the pressure P is low, the λ of Ar and the sputtered-out metal ions are both large, indicating that prior to reaching the target material, the possibility of Ar^+^ ions colliding is small, causing their energy to be high, resulting in more metal ions being bumped out, faster film deposition, and more sputtered metal ions reaching the substrate, which may not have enough time to diffuse sufficiently, leading to poor film quality and low mobility.

When the argon gas flow rate increases, the P gets larger and the λ of Ar and the sputtered-out metal ions becomes smaller, indicating that before arriving at the target material, the Ar^+^ ions may be hit heavily and become low-energy ions, causing fewer metal ions being bombed out, slower film deposition, and not so many sputtered metal ions reaching the substrate, which is beneficial for their diffusion, finally resulting in good film quality and high mobility [31].

However, when the argon gas flow rate is too large, the λ becomes too small, indicating scattering effects between particles enhance [32], which means that both the Ar^+^ ions bombing the target material and the sputtered-out metal ions reaching the substrate are low-energy, causing slower film deposition and less dense the films, finally bringing out inferior film quality and decreased mobility.

Therefore, precise control of carrier concentration is essential to balance mobility and scattering effects. The IGZTO thin films exhibited optimal electrical performance at an argon gas flow rate of 50 sccm.

#### 3.3.3. The Effect of ToSD

Figure 6c is the electrical properties of IGZTO thin films prepared at different ToSDs. When the ToSD was 50 mm, the short distance between the target and substrate reduced the probability of collisions between target particles and argon atoms. This led to a higher deposition rate and an increased concentration of oxygen vacancies in the film. However, the sputtered particles arriving at the glass substrate had higher energies, which caused re-sputtering of the already deposited high-quality film. Some of these energetic particles were implanted into the film, disrupting its structural integrity and creating numerous defects. These defects formed defect energy levels within the bandgap, trapping electrons [33]. As a result, the IGZTO thin films exhibited a low Hall mobility of 27.27 cm^2^/Vs, a carrier concentration of 3.32 × 10^19^ cm^−3^, and a relatively high resistivity of 0.76 × 10^−2^ Ω·cm.

When the ToSD increased from 50 mm to 60 mm, the deposition rate decreased, and the concentration of oxygen vacancies reduced. The increased probability of collisions between target particles and argon atoms led to energy loss in the sputtered particles. The energy of the particles arriving at the glass substrate was reduced to an optimal level for film particle migration [34]. This resulted in the formation of IGZTO thin films with uniform grains and high density, reduced grain boundaries and defects, and improved electrical properties. The Hall mobility increased to 29.91 cm^2^/Vs, the carrier concentration rose to 3.66 × 10^19^ cm^−3^, and the resistivity decreased to 0.54 × 10^−2^ Ω·cm.

Further increasing the ToSD from 60 mm to 70 mm increases the probability of collision at a constant energy, indirectly leading to a decrease in the mean free path of sputtered particles [35]. The increased probability of collision with argon ions further reduced the energy of the sputtered atoms arriving at the glass substrate. This resulted in smaller grains, increased grain boundaries, and higher scattering probabilities for carriers. Additionally, the reduced deposition rate led to lower film density and uniformity. Consequently, the Hall mobility decreased to 26.98 cm^2^/Vs, the carrier concentration dropped to 2.93 × 10^19^ cm^−3^, and the resistivity increased to 0.57 × 10^−2^ Ω·cm.

In summary, the electrical properties of IGZTO thin films were optimized at a ToSD of 60 mm, where the balance between particle energy, deposition rate, and film quality was most favorable.

#### 3.3.4. The Effect of Film Thickness

Figure 6d illustrates the variation in resistivity, Hall mobility, and carrier concentration with thickness in IGZTO thin films. As the film thickness increased from 100 nm to 500 nm, the carrier concentration rose from 2.93 × 10^19^ cm^−3^ to 4.10 × 10^19^ cm^−3^, and the Hall mobility increased from 27.71 cm^2^/Vs to 34.10 cm^2^/Vs, while the resistivity decreased gradually from 0.82 × 10^−2^ Ω·cm to 0.45 × 10^−2^ Ω·cm. These changes indicate a significant improvement in the electrical properties of the films with increasing thickness. This enhancement is primarily attributed to the increased grain size and improved crystallinity of the films as thickness increases, leading to higher uniformity and density. These factors collectively improve film quality and reduce the number of defects, thereby enhancing electrical performance.

For instance, when the film thickness increased from 100 nm to 200 nm, a substantial improvement in electrical properties was observed. At 100 nm, the film exhibited poor crystallinity, a higher density of grain boundaries, and lower uniformity and density, resulting in a higher number of defects and inferior electrical properties (carrier concentration of 2.93 × 10^19^ cm^−3^, Hall mobility of 27.71 cm^2^/Vs, and resistivity of 0.82 × 10^−2^ Ω·cm). In contrast, at 200 nm, the film’s uniformity and density increased significantly, reducing the number of defects and leading to a marked improvement in carrier concentration (3.66 × 10^19^ cm^−3^) and Hall mobility (29.91 cm^2^/Vs) and a substantial decrease in resistivity (0.54 × 10^−2^ Ω·cm).

As the thickness continued to increase beyond 200 nm, the density and crystallinity of the films further improved, resulting in higher carrier concentrations and Hall mobilities and continued reduction in resistivity. However, for thicker films, the increase in interface defects and surface roughness may introduce additional scattering centers [36], leading to a diminished rate of improvement in electrical properties. Consequently, the trend in electrical performance enhancement became more gradual and approached a plateau.

#### 3.3.5. Electric Properties Comparison of IGZTO and Previous IGZO or ITZO Films

Table 2 presents a comparison of the electrical properties of IGZTO thin films prepared by magnetron sputtering at a sputtering power of 90 W, an argon flow rate of 50 sccm, a ToSD of 60 mm, and a thickness of 200 nm with those of previously reported IGZO and ITZO thin films. As shown in Table 2, the IGZTO films prepared in this experiment have lower resistivity and higher average visible transmittance and Hall mobility than IGZO films. In addition, the Hall mobility and average visible transmittance of the IGZTO films prepared in this experiment are higher than that of ITZO films; the carrier concentration, average visible transmittance and resistivity are better than that of IGZTO films prepared by the sol–gel method; and the Hall mobility is better than that of IGZTO films prepared by the magnetron sputtering method. The method of preparation and the reproducibility of the results are better than that of Ga: IZO films prepared by magnetron co-sputtering, which proves that the IGZTO films prepared in this experiment have excellent comprehensive performance.

### 3.4. XPS Analysis of Element Content and Oxygen Vacancies

#### 3.4.1. Element Content of the IGZTO Thin Films

**Figure 7:**
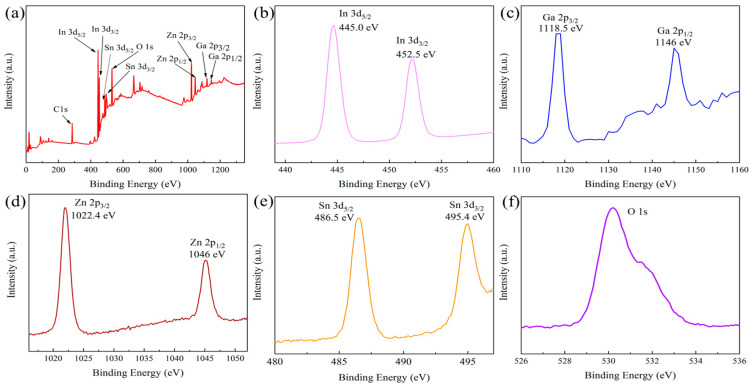
High-resolution XPS spectra of a 200 nm-thick IGZTO thin film prepared at a sputtering power of 90 W, argon gas flow rate of 50 sccm, and ToSD of 60 mm: (**a**) survey spectrum, (**b**) In 3d, (**c**) Ga 2p, (**d**) Zn 2p, (**e**) Sn 3d, and (**f**) O 1s.

Figure 7 presents the XPS spectra of IGZTO thin films deposited at a sputtering power of 90 W, argon gas flow rate of 50 sccm, ToSD of 60 mm, and thickness of 200 nm. The spectra include (a) the survey spectrum and high-resolution spectra of (b) In 3d, (c) Ga 2p, (d) Zn 2p, (e) Sn 3d, and (f) O 1s. All XPS spectra were calibrated using the C 1s peak at 284.8 eV.

As analyzed from Figure 7a, the atomic ratios of metal elements bonded with oxygen are as follows: In 3d is 14.31 at.%, Ga 2p is 5.17 at.%, Zn 2p is 20.32 at.%, Sn 3d is 4.71 at.%, and O is 55.48 at.%. These ratios slightly deviate from the target material composition of In:Ga:Zn:Sn:O = 1:0.5:1.5:0.25:x. This discrepancy is mainly due to the different sputtering yields of various elements during the sputtering process (the sputtering yield of lighter elements, such as oxygen, is usually higher than that of heavier elements like indium, gallium, zinc, and tin). This difference in sputtering yields leads to variations in the atomic ratios of elements in the deposited thin films [38,40].

The results in Figure 7b–e show that the binding energies of In 3d_5/2_ and In 3d_3/2_ are approximately 445 eV and 452.5 eV, respectively, indicating that In exists in the IGZTO films in the In^3+^ oxidation state. The binding energies of Ga 2p_3/2_ and Ga 2p_1/2_ are approximately 1118.5 eV and 1146 eV, respectively, corresponding to Ga in the Ga^3+^ state. The binding energies of Zn 2p_3/2_ and Zn 2p_1/2_ are approximately 1022.4 eV and 1046 eV, respectively, which are attributed to Zn in the Zn^2+^ state. The binding energies of Sn 3d_5/2_ and Sn 3d_3/2_ are approximately 486.5 eV and 495.4 eV, respectively, indicating that Sn is in the Sn^4+^ state [17].

The O 1s spectra are demonstrated in Figure 7f, from which it shows that the binding energies of O 1s are mainly located in the relatively large range of 529~533 eV, and there are at least two peaks. A detailed analysis and discussion of O 1s spectra is given in Section 3.4.3.

#### 3.4.2. Comparison of Zinc Interstitials in Different Samples

**Figure 8:**
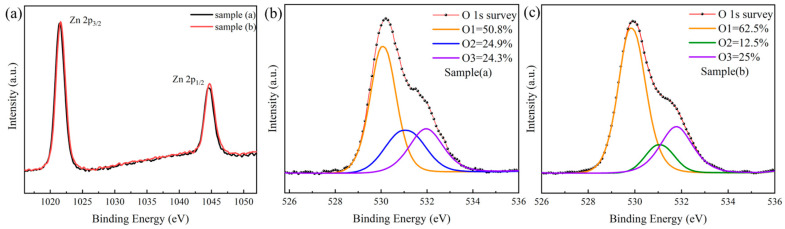
(**a**): XPS Zn 2p spectra. (**b**) and (**c**): XPS O 1s spectra of two IGZTO films prepared with different parameters: sample (a)—90 W, 50 sccm, 60 mm, thickness 200 nm; sample (b)—90 W, 20 sccm, 60 mm, thickness 200 nm.

It is known that electrons come from not only oxygen vacancies but also zinc interstitials in ZnO, so how about in our IGZTO films? In order to answer this question, two IGZTO film sample were prepared: sample (a) is deposited at 90 W, 50 sccm, 60 mm, and 200 nm, and sample (b) is grown at 90 W, 20 sccm, 60 mm, and 200 nm. Their Zn 2p and O1s spectra were measured by XPS, and the results are given in Figure 8.

From Figure 8a, it revealed that the binding energies of the Zn 2p orbitals of our two samples are located at 1022.4 eV (Zn 2p_3/2_) and 1046.0 eV (Zn 2p_1/2_), which are not in accordance with the standard values of about 1021.4 eV (Zn 2p_3/2_) and 1044.5 eV (Zn 2p_1/2_) of intrinsic ZnO [41], which indicates that the Zn 2p binding energies of our samples have a positive shift of 1.0 eV (Zn 2p_3/2_) and 1.5 eV (Zn 2p_1/2_), respectively. The shift of the binding energy means there is a change in the local electronic environment of Zn atoms; i.e., some Zn defects occur. It is reported that the Zn interstitials lead to the Zn 2p_3/2_ binding energy shifting toward higher energies (about 1022.0 eV or higher) [42], indicating that interstitial Zn does exist in the IGZTO films. However, by carefully comparing the XPS high-resolution maps of Zn 2p for samples (a) and (b) under different process parameters, the Zn 2p_3/2_ peaks are found to almost overlap, indicating that the variation of preparation process parameters has less effect on the Zn interstitials. In other word, the different electrical properties of our IGZTO oxide films are not directly dependent on the concentration of Zn interstitials.

#### 3.4.3. Comparison of Oxygen Vacancies in Different Samples

The XPS O1s spectra of the two IGZTO film samples are shown in Figure 8b,c, respectively. The O 1s XPS spectrum of the IGZTO films can be decomposed well into three Gaussian–Lorentzian components centered at 530.0 ± 0.2 eV (O1), 531.0 ± 0.1 eV (O2), and 532.0 ± 0.3 eV (O3). The O1 peak represents oxygen atoms bonded in the lattice [43,44], such as Zn-O and In-O [45]. The O2 peak is associated with oxygen ions in oxygen-deficient regions of the matrix and is related to the concentration of oxygen vacancies (Vo) [46]. The O3 peak is attributed to adsorbed oxygen species on the film surface [47], such as O_2_, H_2_O, –CO_3_, and –OH groups, as well as interstitial oxygen [48]. In metal oxide semiconductors, oxygen vacancy act as donors, and thus, the electrical properties are closely related to the oxygen vacancy concentration [49,50].

From the O 1s XPS spectrum of the IGZTO sample (a), displayed in Figure 8b, the calculated area percentage of the three O peaks are 50.8 (Area%), 24.9 (Area%), and 24.3 (Area%) for the O1, O2, and O3 peak, respectively. The dominant O1 peak indicates a high concentration of O^2−^ ions bonded in the IGZTO lattice. The relatively high O2 peak, second only to O1, suggests there is a significant concentration of oxygen vacancies.

Similarly, the area percentages of O1, O2, and O3 peaks of sample (b) shown in Figure 8c are calculated. The area percentage of the three O peaks of the two samples are listed in Table 3.

From Table 3, it shows that the percentage of O2 peak area is 24.9% and 12.5% for sample (a) and (b), respectively, indicating that the oxygen vacancy concentration in sample (a) is higher than sample (b), which is consistent with the carrier concentration of sample (a), 3.66 × 10^19^ cm^−3^, being larger than that of sample (b), 5.0 × 10^18^ cm^−3^. As a result, the electrical properties (Hall mobility 29.91 cm^2^/Vs, resistivity 0.54 × 10^−2^ Ω·cm) of the sample (a) are better than those of sample (b) (Hall mobility 21.50 cm^2^/Vs, resistivity 5.69 × 10^−2^ Ω·cm).

In a short word, the electrical properties of oxide films are mainly ascribed to the concentration of oxygen vacancies, which is in agreement with the viewpoint reported by Sun et al. that oxygen vacancies are intrinsic donor defects that facilitate n-type conductivity in films [51]. In order to achieve the desired electrical properties of IGZTO thin films, one should obtain a suitable oxygen vacancy concentration by adjusting and optimizing the preparation parameters of the films.

## 4. Conclusions

In this study, four series of IGZTO thin films were prepared by RF magnetron sputtering, among which three are all 200 nm thick but deposited under different sputtering powers, argon gas flow rates, or ToSD, and the fourth is with different thicknesses of 100–500 nm. The effects of fabrication parameters (sputtering power, argon gas flow, and ToSD) and film thickness on the microstructure, optical, and electrical properties of IGZTO thin films were investigated, and the conduction mechanisms were also discussed briefly. The obtained main results are as follows:

(1) All the obtained IGZTO thin films are amorphous in structure with stable phase characteristics and have a smooth and uniform surface with a low roughness (RMS of 0.441 nm, RA of 0.332 nm).
(2) All the obtained IGZTO thin films are transparent, demonstrating good average visible light transmittance (89.02~90.69%), low Urbach energy (152~353 meV), and an optical bandgap of 3.47~3.56 eV.
(3) The 200 nm thick IGZTO film obtained at 90 W sputtering power, 50 sccm argon gas flow rate, 60 mm target-to-substrate distance exhibits optimal electrical properties with a carrier concentration of 3.66 × 10^19^ cm^−3^, Hall mobility of 29.91 cm^2^/Vs, and resistivity of 0.54 × 10^−2^ Ω·cm.
(4) XPS analysis revealed that Zn interstitials exist in the IGZTO films, but its content varies little in different samples, means, Zn interstitials are not the origin of the different electrical properties of various IGZTO films.
(5) XPS analysis indicated that O atoms have three states in IGZTO films: oxygen atoms bonded with metal in the lattice (such as In–O, Ga–O), oxygen vacancies, and adsorbed oxygen species on the surface or interstitial oxygen. Among them, the oxygen-deficient defects act as donors, providing the main carrier. In other word, the electrical properties of IGZTO films are mainly ascribed to the concentration of oxygen vacancies.

In summary, the obtained IGZTO films have stable structures and excellent optoelectronic properties. These findings provide a helpful reference for the property modulation of IGZTO films and valuable insight for the potential applications of IGZTO films in fields related to transparent conductive optoelectronic devices such as TFTs.

## Figures and Tables

**Table 1:** Deposition conditions for four series of IGZTO thin films.

Variable Series		Sputtering Power (W)	Argon Flow (sccm)	ToSD (mm)	Film Thickness (nm)
Same thickness	Sputtering power	60, 70, 80, 90, 100, 110	30	60	200
	Argon flow	90	20, 30, 40, 50, 60	60	200
	ToSD	90	50	50, 55, 60, 65, 70	200
Different thickness		90	50	60	100, 200, 300, 400, 500

**Table 2:** Comparison of electrical properties of our IGZTO and several representative IGZO, IGZTO, and ITZO films that were reported previously.

Thin Film	Method	Carrier Concentration (cm^−3^)	Hall Mobility (cm^2^/Vs)	Electrical Resistivity (Ω·cm)	Average Visible Light Transmittance	E_g_ (eV)	Ref.
IGZO	MS *	/	7.7	/	/	/	[7]
IGZTO	MS	/	12.2	/	/	/	[7]
Ga: IZO	Mc-S *	1.8 × 10^20^	29	3.0 × 10^−3^	86.5%	3.40	[10]
IGZTO	Sol–gel	3.48 × 10^15^	65	27.78	89%	3.66	[17]
IGZO	SP *	/	/	1.05 × 10^−2^	>70%	3.39	[37]
IGZO	MS	/	/	3.4 × 10^−2^	91.93%	3.79	[38]
ITZO	MS	1.27 × 10^20^	24.31	/	89.87%	3.74	[39]
IGZTO [This work]	MS	3.66 × 10^19^	29.91	0.54 × 10^−2^	90.21%	3.54	/

* Note: MS stands for magnetron sputtering, Mc-S stands for magnetron co-sputtering, and SP stands for spray pyrolysis.

**Table 3:** Location, meaning, and percentage of O1, O2, and O3 peaks in two IGZTO film samples.

Peak Name	Binding Energy (eV)	Meaning	Area Percentage of Peak (Area%)	
			Sample (a)	Sample (b)
O1	530.0 ± 0.2	Oxygen atoms bonded in the lattice [43,44], such as Zn-O and In-O [45].	50.8	62.5
O2	531.0 ± 0.1	Oxygen vacancies (Vo) [46].	24.9	12.5
O3	532.0 ± 0.3	Adsorbed oxygen species on the film surface [47], such as O_2_, H_2_O, –CO_3_, and –OH groups [48].	24.3	25
Carrier concentration	/	/	3.66 × 10^19^ cm^−3^	5.0 × 10^18^ cm^−3^

Note: The deposit condition of two film samples: (a) 90 W, 50 sccm, 60 mm, 200 nm thick; (b) 90 W, 20 sccm, 60 mm, 200 nm thick.

## Data Availability

The original contributions presented in this study are included in the article. Further inquiries can be directed to the corresponding authors.
